# Nine Forward–Backward Translations of the Hopkins Symptom Checklist-25 With Cultural Checks

**DOI:** 10.3389/fpsyt.2021.688154

**Published:** 2021-08-12

**Authors:** Patrice Nabbe, Jean Yves Le Reste, Morgane Guillou-Landreat, Radost Assenova, Djurdjica Kasuba Lazic, Slawomir Czachowski, Stanislava Stojanović-Špehar, Melida Hasanagic, Heidrun Lingner, Ana Clavería, María Rodríguez-Barragán, Agnieszka Sowinska, Stella Argyriadou, Charileos Lygidakis, Bernard Le Floch, Tristan Montier, Harm Van Marwijk, Paul Van Royen

**Affiliations:** ^1^Department of General Practice, EA 7479 Soins primaires, Santé publique, Registre des cancers de Bretagne Occidentale, Université de Bretagne Occidentale, Brest, France; ^2^Department of Addictology, EA 7479 Soins primaires, Santé publique, Registre des cancers de Bretagne Occidentale, Université de Bretagne Occidentale, Brest, France; ^3^Department of Urology and General Medicine, Faculty of Medicine, Medical University of Plovdiv, Plovdiv, Bulgaria; ^4^Department of Family Medicine “Andrija Stampar, ” School of Public Health, School of Medicine, University of Zagreb, Zagreb, Croatia; ^5^Department of Clinical Psychology and Neuropsychology, Nicolaus Copernicus University, Torun, Poland; ^6^Health Care Studies, University “Djemal Bijedic, ” Mostar, Bosnia and Herzegovina; ^7^Centre for Public Health and Healthcare, Hannover Medical School, Hanover, Germany; ^8^Xerencia Xestión Integrada de Vigo, Servizo Galego de Saúde, Instituto de Investigación Sanitaria Galicia-Sur, Red de Investigación en Actividades Preventivas y de Promoción de la Salud, Vigo, Spain; ^9^Centro de Atención Primaria La Mina, Gerencia Territorial de Atención Primaria de Barcelona, Instituto Catalán de la Salud, Sant Adrià de Besòs, Barcelona, Spain; ^10^Fundación Instituto Universitario de Investigación en Atención Primaria de Salud Jordi Gol i Gurina (IDIAP Jordi Gol), Barcelona, Spain; ^11^Universidad Autónoma de Barcelona, Barcelona, Spain; ^12^Department of Experimental Linguistics, Nicolaus Copernicus University, Torun, Poland; ^13^Escuela de Inglés, Universidad Catolica del Norte, Antofagasta, Chile; ^14^The Greek Association of General Practitioners (ELEGEIA), Thessaloniki, Greece; ^15^Department of Behavioral and Cognitive Sciences, University of Luxembourg, Esch-sur-Alzette, Luxembourg; ^16^INSERM, Etablissement Français du Sang, UMR 1078, Génétique, Génomique Fonctionnelle et biotechnologies, Univ Brest, Brest, France; ^17^Service de Génétique Médicale et Biologie de la Reproduction, CHRU de Brest, Brest, France; ^18^Department of Primary Care and Public Health, Brighton and Sussex Medical School, University of Brighton, Brighton, United Kingdom; ^19^Department of Family Medicine, Faculty of Medicine and Health Sciences, University of Antwerp, Antwerp, Belgium

**Keywords:** depression, Hopkins Symptom Checklist-25, depressive disorder, HSCL-25, diagnostic tool

## Abstract

**Introduction:** The Hopkins Symptom Checklist-25 (HSCL-25) is an effective, reliable, and ergonomic tool that can be used for depression diagnosis and monitoring in daily practice. To allow its broad use by family practice physicians (FPs), it was translated from English into nine European languages (Greek, Polish, Bulgarian, Croatian, Catalan, Galician, Spanish, Italian, and French) and the translation homogeneity was confirmed. This study describes this process.

**Methods:** First, two translators (an academic translator and an FP researcher) were recruited for the forward translation (FT). A panel of English-speaking FPs that included at least 15 experts (researchers, teachers, and practitioners) was organized in each country to finalize the FT using a Delphi procedure.

**Results:** One or two Delphi procedure rounds were sufficient for each translation. Then, a different translator, who did not know the original version of the HSCL-25, performed a backward translation in English. An expert panel of linguists compared the two English versions. Differences were listed and a multicultural consensus group determined whether they were due to linguistic problems or to cultural differences. All versions underwent cultural check.

**Conclusion:** All nine translations were finalized without altering the original meaning.

## Introduction

How to manage people with depression in primary care is a growing challenge worldwide. Indeed, Family practice physicians (FPs) are at the frontline, while secondary care services are increasingly under threat (1–4). Depression manifests (for laypersons) itself in various ways: (i) as a syndromic “disorder” in which contextual distress, anxiety, and somatoform disorders overlap; (ii) as a suffering that is difficult to express, acknowledge, and discuss; and (iii) as a long-term condition with subjective and objective features that can be measured (5). Due to these inter-individual variabilities, FPs may experience difficulties in detecting depression and may easily misjudge the symptoms and their intensities, if they do not use formal instruments (6, 7). Moreover, the depression incidence and prevalence rates differ widely in family practice, due to complex contextual variations, differences in healthcare systems, concepts of disorder, objectives, and practices, as well as cultural variations in symptom expression (8, 9). These difficulties may lead to inappropriate care and potential side effects due to drugs' use as well as public health issues (10–12). A short discussion of the results obtained using a relevant questionnaire is often the first step toward an open dialogue with the patient.

Collaborative primary care mental health models can improve the management of patients with depression. To this aim, the European General Practice Research Network (EGPRN) developed a collaborative research agenda (13). Specifically, the EGPRN adopted a standardized methodology in which European FPs experts from different healthcare systems and who speak different languages and have different cultural references set up an established consensus procedure to identify reliable, standardized, efficient, and ergonomic tools for depression assessment that take into account cultural and linguistic differences (14–17). These tools need to be accepted by both FPs and psychiatrists to improve collaboration (18). They must be feasible in the FP's surgery, in primary or psychiatric care, and also suitable for research purposes (19). Finally, they must be validated and reliable.

A handbook was developed to guide the selection of a single tool that would be then translated into different languages, using a forward and backward translation procedure (inspired by Brislin's translation model). This is a consensual procedure that has been used in other cross-cultural studies (20–22). At each step, the key points and purposes were debated and decided by consensus among the involved European experts. First, a systematic literature review, according to the PRISMA criteria, allowed the identification of seven tools that had been validated against a psychiatric examination using the DSM-IV or DSM-5 major depression criteria (23). Then, a consensus procedure (RAND/UCLA Appropriateness Method) led to the selection of one tool on the basis of its effectiveness, reliability, and ergonomics (24): the self-report Hopkins Symptom Checklist-25 (HSCL-25) (23–26). This is a validated, reliable diagnostic tool to assess (27, 28) the presence and severity of anxiety and depression symptoms during the previous week (29, 30). Its specificity compared with clinical interview is robust: between 0.78 to 0.88, the reliability (Alpha de Cronbach) is between 0.87 to 0.97 (31). The HSCL-25 short length self-administered format is perfectly suited for use in busy primary care settings with many competing demands. It may represent a practical instrument to alert FPs to potentially depressive or anxious symptomatology.

A qualitative procedure with the FP's involvement was necessary to obtained that were linguistically and culturally equivalent to the original version, ecologically embedded in primary care.

The objective of the present study was to translate the HSCL-25 into the languages of the different team members, without losing homogeneity, and in a language suitable to the primary care context (22, 32).

## Materials and Methods

**Figure 1:**
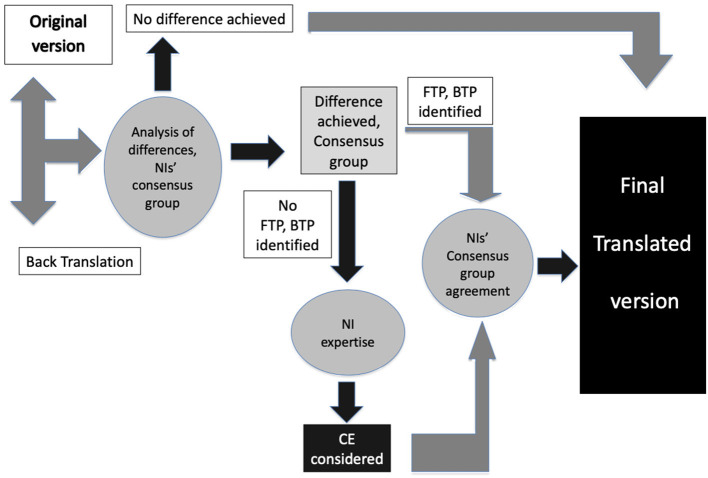
The translation procedure. CE, cultural effect; BTP, backward translation problems; FTP, forward translation problems.

This three-step standardized study included: (i) forward translation (FT), (ii) backward translation (BT), and (iii) cultural check (8, 33, 34) (Figure 1).

The FT was carried out with an incorporated Delphi consensus procedure (35–37). This is a systematic, interactive method that involves a panel of experts using iterative procedures (38) and that allows reaching consensus in a rigorous way (39–41). This process requires:

- Anonymity of participants to ensure response reliability and avoid contamination,
- Iteration, which allows participants to refine their views in the light of the group work progress,
- Feedback control under the investigator's responsibility,
- Statistical aggregation of the group's responses to allow a quantitative and qualitative analysis of data (42–45).

The EGPRN French team ensured that this protocol was followed throughout the process. The FT of the different HSCL-25 items had to be validated daily by the expert panel, composed of EGPRN members, all actively involved in the process.

Briefly, for each language, the National Investigators (NI) selected translators knowledgeable about healthcare terminology to organize two translation (FT and BT) teams who were blind to the other team's work. The FT team included one member of the FP research group and one official translator for each country. The BT team involved one (or two) FPs and one official translator (22).

The NIs also recruited a panel of FP experts in their own countries, anonymized the experts' responses, and allocated an identification number for later identification (42). Initially, 20 to 30 experts were recruited per country to secure the presence of at least 15 participants till the project end. The FP experts were selected using the following inclusion criteria: native of their country of residence and speaking their native language, and fluent in English (32). At least half of them had to be involved in teaching and/or research activities. To assess the panel representativeness of their country FPs, the experts provided the following information: sex, practice type, years of practice, and publication record (46).

According to the Brislin's Guidelines for the Process of Cross-Cultural Adaptation of Self-Report Measures, once the FT was completed, a BT was performed with two goals: (i) to ensure the identification of language issues and (ii) to detect translation problems linked to cultural adaptation issues. Indeed, as translation biases related to cultural aspects of each country were possible, a cultural check was required to ensure homogeneity (17, 20, 33, 34, 47). To this aim, in each country, an FP researcher and a linguist analyzed all BT propositions and compared them with the original HSCL-25 version to establish whether there was any significant difference in terms of meaning. Their report was submitted to a consensus group whose task was to clarify the nature of each FT-BT discrepancy from three problem areas: (i) BT problems were eliminated if the difference was explained by an incorrect BT; (ii) FT problems were defined as an anomaly in transcribing the original English (semantic/idiomatic differences relative to the original English version); and (iii) cultural effects (CE) were considered validated if there was no linguistic problem with the translation, but the item needed to be modified to be understood by the patients in their own “everyday” language (Figure 1).

This led to a linguistically stable, definitive translation that maintained the HSCL-25 meaning (i.e., structure and question order and method of use) for each involved country.

Ethical request: The EGPRN French team was in charge of checking the volunteering process and confirming the absence of potential conflicts of interest for all participants. The Ethics Committee of the approved the whole process.

The EGPRN French team recruited all NIs and obtained their consent, managed the voluntary participation in the study and produced an absence of conflict-of-interest statement.

Each NI asked participants to sign the informed consent.

## Results

### NI Panel Description

The NI panel included 11 NIs (including *n* = 8 women) from eight European countries. They were all FPs, EGPRN members, and fluent in English. Ten NIs practiced in urban areas of more than 5,000 inhabitants and one worked in an urban area with 2,000–5,000 inhabitants. Eight had also teaching duties in addition to being researchers (total number of publications by the panel members: 152). The mean number of years of practice and of research were 21.3 and 12.4 years, respectively. In the panel, two NIs were from two distinct cultural regions of coastal Spain (Catalonia and Galicia), and two were Croats. The other countries were each represented by a single NI (Table 1).

**Table 1:** National investigators' panel.

**Experts**	**Gender**	**Country**	**Academic Status**	**Number of inhabitants**	**Practice type**	**Number of international publications**	**Years of practice**	**Years of research**
9	F	Bulgaria	Teacher/Researcher	>5,000	FP group practice	9	14	12
7	F	Croatia	Teacher/Researcher	>5,000	Alone	6	20	12
8	F	Croatia	Teacher/Researcher	>5,000	FP group practice	18	30	20
11	M	France	Teacher/Researcher	>5,000	FP group practice	11	20	5
5	F	Germany	Researcher	2,000–5,000	Ceased practicing 2 years previously	19	23	5
10	F	Germany	Researcher	>5,000	FP group practice	4	18	7
3	F	Greece	Teacher/Researcher	>5,000	FP and paramedic group practice	14	30	18
4	M	Italy	Researcher	>5,000	FP group practice	23	7	6
6	M	Poland	Teacher/Researcher	>5,000	FP group practice	20	30	12
2	F	Spain (Cataluña)	Teacher/Researcher	>5,000	FP group practice	13	22	25
1	F	Spain (Galicia)	Teacher/Researcher	>5,000	FP group practice	15	20	14

*F, female; M, male; FPs, family practice physicians*.

### Forward Translation

For the Delphi consensus procedure, 14 (Germany) to 31 experts (Spain) were recruited. In compliance with the selection criteria, they were all FPs and fluent in English. The expert panel included 215 FPs (111 men and 104 women). Among them, 20 worked in a city of <2,000 inhabitants, 36 in a city with 2,000–5,000 inhabitants, and 159 in a city with >5,000 inhabitants. Their clinical experience was analyzed according to years of practice (mean: 16.4 years of experience) (Table 2).

**Table 2:** Characteristics of each country expert panel.

	***N* (women)**	**Practice (mean years)**	**Number of inhabitants in the practice area**			**Academic researcher and/or teacher**		**Number of publications**	**Participants in the second Delphi round**
			**<2,000**	**2,000–5,000**	**>5,000**	**Number**	**Experience (mean, years)**		
Bulgaria	22 (13)	20.5	1	5	16	5	5.4	8	No second round
Catalonia	22 (9)	15.7	0	2	20	20	10.5	22	No second round
Croatia	16 (13)	19.2	1	1	14	16	11.5	15	15
France	16 (7)	12.5	1	7	8	15	6.3	11	15
Galicia	20 (6)	22.3	0	0	20	17	13.1	19	20
Germany	14 (8)	16.7	0	3	11	9	10	6	No second round
Greece	26 (13)	10.9	10	9	7	24	5.1	26	15
Italy	18 (6)	17.2	3	2	13	13	14	12	No second round
Poland	30 (18)	11.9	4	6	20	26	13.1	10	No second round
Spain	31 (11)	19.5	0	1	30	27	12	30	No second round
Total	215 (104)	15.55	20	36	178	172	10.1	159	4 Second round

In Poland, Bulgaria, Germany, Spain, and the Catalonia region of Spain, there was only one Delphi round, and two rounds in the other countries. Almost all translation proposals for each item of the HSCL-25 questionnaire were accepted in one round (273/320: 85.3%) (Table 3). The other proposals for which consensus was not reached went through a second round. The NI and the forward official translator synthesized the experts' comments to produce a new translation proposition for the second round.

**Table 3:** Results of the first Delphi round.

**Item/Country**	**Galicia**	**Castile**	**Catalonia**	**France**	**Italy**	**Bulgaria**	**Croatia**	**Greece**	**Germany**	**Poland**
1 Being scared for no reason	C	C	C	C	C	C	C	NC	C	C
2 Feeling fearful	C	C	C	C	C	C	NC	C	C	C
3 Faintness	C	C	C	NC	NC	C	NC	NC	C	C
4 Nervousness	C	C	C	C	C	C	C	C	C	C
5 Heart racing	C	C	C	NC	C	C	C	C	C	C
6 Trembling	NC	C	C	NC	NC	C	C	C	C	C
7 Feeling tense	C	C	C	C	C	C	C	C	C	C
8 Headache	C	C	C	C	C	C	C	C	C	C
9 Feeling panic	C	C	C	NC	C	C	NC	C	C	C
10 Feeling restless	NC	C	C	NC	C	C	NC	C	C	C
11 Feeling low in energy	C	C	C	C	C	C	NC	NC	C	C
12 Blaming oneself	C	C	C	NC	NC	C	C	C	C	C
13 Crying easily	C	C	C	C	C	C	C	NC	C	C
14 Losing sexual interest	C	C	C	NC	C	C	NC	C	C	C
15 Feeling lonely	C	C	C	NC	C	C	NC	C	C	C
16 Feeling hopeless	C	C	C	C	C	C	NC	C	C	C
17 Feeling blue	C	C	C	NC	C	C	NC	C	C	C
18 Thinking of ending one's life	C	C	C	C	C	C	C	NC	C	C
19 Feeling trapped	C	C	C	NC	C	C	C	C	C	C
20 Worrying too much	C	C	C	NC	C	C	NC	NC	C	C
21 Feeling no interest	C	C	C	NC	C	C	NC	NC	C	C
22 Feeling that everything is an effort	C	C	C	C	C	C	C	C	C	C
23 Feelings of worthlessness	C	C	C	NC	C	C	C	NC	C	C
24 Poor appetite	C	C	C	C	C	C	C	NC	C	C
25 Sleep disturbance	NC	C	C	NC	C	C	C	C	C	C
26 Choose the best answer for how you felt over the past week	C	C	C	NC	C	C	C	C	C	C
27 Not at all	C	C	C	C	NC	C	C	C	C	C
28 A little	C	C	C	NC	C	C	C	C	C	C
29 Quite a bit	C	C	C	C	C	C	C	C	C	C
30 Extremely	C	C	C	C	C	C	C	C	C	C
31 The HSCL-25 score is calculated by dividing the total score (sum score of items) by the number of items answered (ranging between 1.00 and 4.00). It is often used as the measure of distress.	C	C	C	NC	NC	C	C	C	C	C
The patient is considered as a “probable psychiatric case” if the mean rating on the HSCL-25 is ≥1.55.										
32 A cut-off value of ≥1.75 is generally used for diagnosis of major depression defined as “a case in need of treatment.” This cut-off point is recommended as a valid predictor of mental disorder as assessed independently by clinical interview, somewhat depending on diagnosis and gender.	C	C	C	NC	C	C	C	C	C	C
The administration time of HSCL 25 is 5–10 min										

*C, consensus; NC, no consensus*.

### Some Translation Issues Required a Second Proposal and Another Delphi Round

In Croatian, eleven proposals were rejected in the first round. For example, for item #17 (“Feeling blue”), the first proposal was “Bili ste tužni,” which was considered to be too focused on melancholia, and was modified to “Bili ste sjetni,” closer to the concept of sadness. All new proposals were accepted during the second round.

As a German version of the HCL-25 was already available, the German NIs proposed that their expert panel would discuss this version, without producing a new FT. All items were accepted in the first Delphi round. At this step, the German NIs stopped the procedure. No cultural check was performed.

Nine Greek proposals were rejected in the first round. For example, for item #1 (“Being scared for no reason”): the first proposal “Είμαι τρομοκρατημένος χωρίς αιτία” was considered too strong. Consensus was reached on the second proposal: “Είμαι τρομαγμένος χωρίς αιτία.” All new proposals were accepted during the second round.

In the French translation, consensus was not reached on 18 proposals in the first round and needed further specification in the second round. For example, for item #25 (“Sleep disturbance”), the first proposal was “Vous n'arrivez pas à dormir” that was modified to “Votre sommeil était perturbé,” closer to the English word: “disturbance.” All new proposals were accepted during the second round.

In the Italian translation, consensus was not reached on five proposals during the first round. For example, for item #5 (“Heart racing”), the first proposal “Avere tachicardia” was considered too focused on clinical symptoms and was modified to “Sentire il cuore battere veloce,” which was more familiar according to the reviewers. All new proposals were accepted during the second round.

In the Spanish Galician translation, consensus was not reached on three proposals in the first round. For example, for item #6 (“Trembling”), the first proposal was “Trema,” the present indicative of the verb “Tremar.” The second proposal was “Ten tremores” and was accepted in the second round. All new proposals were accepted during the second round.

### Backward Translation and Cultural Check

The initial instructions, the 25 items, the quotation and the explanatory sentences were all back-translated into English by the BT team. In total, 36 propositions were analyzed. All BTs were compared linguistically to the original. Differences were noted for submission to the NIs and the consensus group. Three consensus group meetings were necessary with national feedback between each. The main adaptations, produced as a result of national feedback and the consensus resulting from the cultural check, are described below.

#### By Languages and Language Groups

Croatia: 8 items were different (2 were BT problems, and 8 required a cultural adaptation).

The main cultural aspect was the use of the present perfect, which is a tense of state and not of action, commonly employed in daily life. Therefore, in items #2, 7, 9, and 10, “feeling” was replaced by “you have been.” Only one item seemed to be stronger than in the original version. Indeed, “Faintness,” was replaced by “Weakness,” but in Croatian this is equivalent to faintness.

Bulgaria: 3 items were different (2 were BT problems, and 1 required a cultural adaptation).

“Feeling low in energy” became “A sense of low energy.” Overall, the Bulgarian translation was the most stable among the three Slavic languages.

Poland: 13 items were different (7 were BT problems, and 6 required a cultural adaptation).

Most problems resulted from a conceptual issue. For instance, in Polish, “Heart racing” became “Palpitations,” “Trembling” became “Tremors,” and “An effort” was translated into “A burden.” “Headache” was translated into “Headaches” in Polish for grammatical reasons.

In all three slavic languages (Croatian, Bulgarian, and Polish), “Feeling restless” was translated into “Anxiety” because there is no equivalent word to express these ideas. A word-by-word translation, in that case, was impossible.

For the Greek language, the translation was mainly based on an adaptation according to gender. The experts concluded that there was a general CE affecting all parts of the scale. However, no real difference in meaning was detected, and the Greek HSCL-25 scale remained stable relative to the original.

France: 5 items were different (4 were BT problems, and 1 required a cultural adaptation).

For the French scale, the present tense is normally used in everyday language. However, the past tense was used in the FT. In everyday life French, the past tense is considered an older, upper-class language style. Therefore, all tenses were modified. For instance, “Tout était un effort pour vous” became “Tout est un effort pour vous” in the final version.

Italy: 7 items were different (6 were BT problems, and 1 required a cultural adaptation).

In the Italian scale, the male plural form was used because this is the usual way of speaking/writing; the translation had to be modified according to gender.

Spain: 6 items were different (1 was a BT problem, and 5 required a cultural adaptation).

“Feeling no interest” was translated in “No siente interes por nada” in standard Spanish, and “Worthless feeling” became “Feeling useless.” However, in Standard Spanish, “inutil” means also “worthless.”

Catalonia: 7 items were different (4 were BT problems, and 3 required a cultural adaptation).

Galicia: 5 items were different (1 was a BT problem, and 4 required a cultural adaptation).

In the Galician scale, item #14 “Losing sexual interest,” was translated into “Loss of sexual interest” that expresses a state, and not an action (the original English version); however, the local experts considered it a normal way of speaking/writing in that language.

In the Galician and Catalan translations, “Blame oneself” turned into “Blame yourself” in the BT because the term “oneself” is not commonly employed.

For the Hispanic languages, the translation had to be modified according to gender. The item “Faintness” was translated into “Weakness” (e.g., “Debilidad,” “Debilitat,” and “Debilidade” in standard Spanish, Catalan and Galician respectively). Similarly, the item “Heart racing” was translated into “Palpitations” (i.e., “Palpitaciones” and “Palpitacions” in the standard Spanish and Galician versions).

#### For All of Languages

Item #17 “Feeling Blue” generated a CE in six of the nine languages. A word-by-word rendition was impossible and required a cultural adaptation.

Items #15 “Feeling lonely,” #18 “Thinking of ending one's life,” #19 “Feeling trapped” and #25 “Sleep disturbance” remained stable after the BT.

Concerning the scale instructions and the quotation question, the BT was different from the original version in nine items, except the explanation concerning the time required to fill in the scale. Many translation problems were related to “cultural” effects. For example: in French, some terms were replaced by typical expressions commonly employed in questionnaires: e.g., “pencil-and-paper” was translated into “auto questionnaire” and “Not at all” by “Pas du tout d'accord.”

Interestingly, there were translation similarities (often with stronger meanings or medical connotations) not only among languages belonging to the same linguistic group, but also among languages from different groups. The best example concerns item #3 “Faintness” that was translated into “Weakness” in Catalan, Standard Spanish, Galician, and also in Croatian, a term with a more prosaic than medical connotation.

At the end of the cultural analysis, the consensus group finally concluded that the meaning was not changed, and the translation was finalized in all nine languages (see Tables 4–6).

**Table 4:** Final translation of the HSCL-25 in nine European languages: items 1–25.

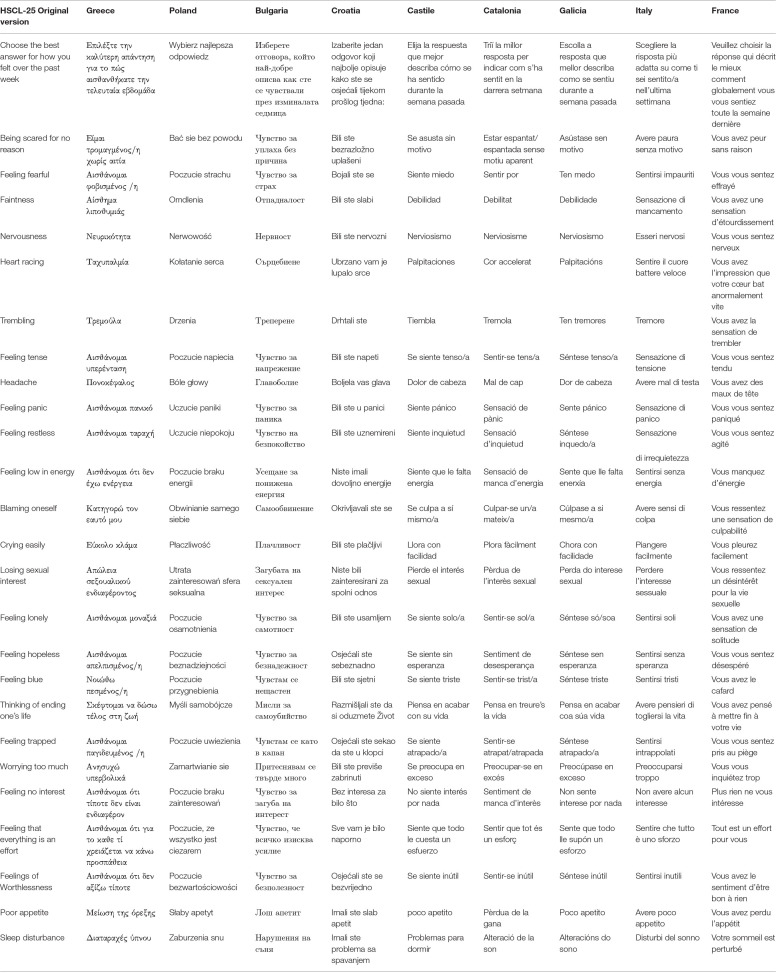

**Table 5:** Final translation of the HSCL-25 in nine European languages: scale instructions.

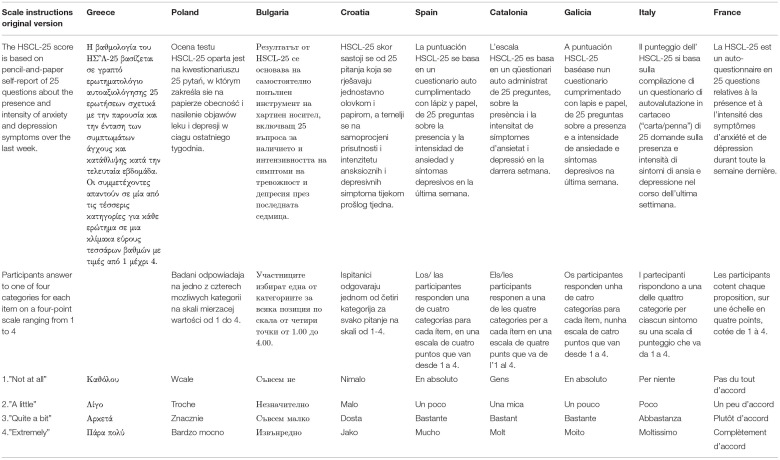

**Table 6:** Final translation of the HSCL-25 in nine European languages: general instructions.

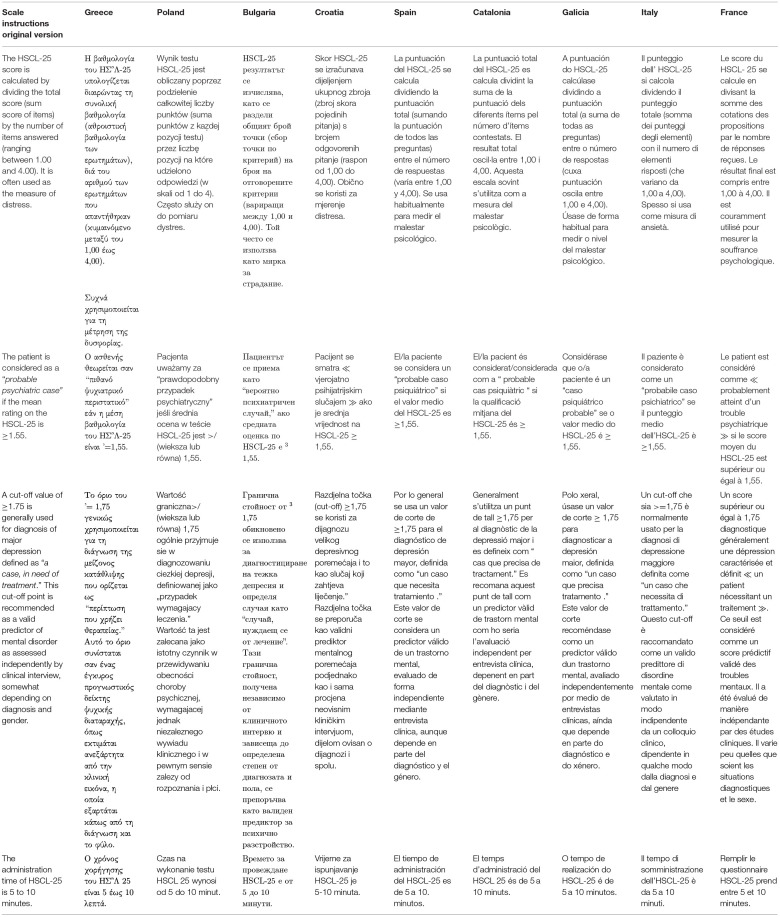

## Discussion

Using a three-step qualitative procedure, ecologically embedded in primary care, nine consensual translations of the HSCL-25 were obtained that were linguistically and culturally equivalent to the original version, in three language families (Hellenic, Slavic, and Romance). A German version already existed. The aim of this procedure was to meticulously track inconsistencies between local translations that could lead to misinterpretation. This methodical and transcultural validation ensured the transfer of the same content from one language to another and its reliability (17, 47).

The Greek translation remained the most stable, followed by Bulgarian. Item #17, “Feeling blue” was the most challenging to translate, followed by item #3 “Faintness” and item #5 “Heart racing.” Some scales needed adaptations in terms of tense (French, Croatian) and in terms of gender (Greek, Italian, and Hispanic languages).

### Research and Teaching Implications

Translation remains the most crucial step in the adoption of an instrument developed in another nation using a different language. Errors in translation may distort the original intent of the instrument, thus compromising its validity and reliability (48). Semantic issues might affect comparability in international studies because the same word is interpreted differently across countries and cultures (49, 50). Moreover, some terms and concepts may not exist in other languages or may have additional connotations that backward translations do not always reveal. Challenges arise not only because of the word-to-word literal translation, but also because of the linguistic form of the language, such as tone and syntax (51).

These nine translations of the HSCL-25 are now linguistically similar, in terms of meaning, compared to the original version. However, they need further testing because this first step is not sufficient to complete the task of translating them and supporting their cross-cultural validity. The external and internal validity of each version has to be evaluated to ensure that their reliability is comparable with that of the original version. This will be achieved through quantitative studies in primary care daily practices (52).

In most European countries, FPs can now use this tool for family practice research studies and for assessing depression severity in their patients. The use of such a shared tool may have a great impact on the feasibility of future research on depression in primary care. It will facilitate data comparison among European countries and consequently it will allow statistical reviews on depression epidemiology and symptoms throughout Europe. The use of the same instrument can support the conceptualization of the studied phenomenon across different studies, and the findings can then be compared (21).

## Limitations

A key point of this study was the FPs' involvement in the translation to reduce the selection bias and to ensure the sample quality nevertheless as in all formalized expert consensus procedure a selection bias of the experts remained possible. Our experts' sample was constructed purposively and if we did our best to avoid a selection bias it remained possible. As described by many translators when discussing scientific translation work, a “specialist” in the field (e.g., primary care daily practice in this case) should take a last look at the translation (20, 53, 54) and become the main arbiter of the quality of the final translation (55). Thus, specific attention was paid in choosing FP researchers and certified bilingual translators with sufficient knowledge of healthcare terminology a selection bias was still possible.

The cultural control check was as consistent as possible. It involved a careful step-by-step analysis to prevent confusion bias and linguistic problems. The formalized consensus method allowed the gradual evaluation of each item to strengthen the accuracy of the validated translations and designing the end-result. Nevertheless, an information or a confusion bias remained possible. Our results should be interpretated in the light of these limitations.

## Conclusion

A translation of the HSCL-25 in which homogeneity is ensured is now available for Spain and its culturally distinct regions of Galicia and Catalonia, and also for France, Greece, Italy, Poland, Bulgaria, and Croatia. It is now ready to be tested in actual and representative primary care populations to further validate its test-parameters.

## Data Availability Statement

The raw data supporting the conclusions of this article will be made available by the authors, without undue reservation.

## Ethics Statement

The studies involving human participants were reviewed and approved by CPP (Protection of Persons Committee) of the University Hospital of Brest. Reference CPP: CPP Ouest VI 872; Study ID RCB: n°2014-A01790-47. The patients/participants provided their written informed consent to participate in this study.

## Author Contributions

PN designed the study, collected data, led meetings, drafted the article, and submitted it for publication. JL designed the study, collected data, attended meetings, and reviewed the article. MG-L and BL reviewed the article. RA, DK, SC, MH, HL, AC, MR-B, AS, SA, and CL participated as national investigator. SS-S participated as co-national investigator. TM reviewed the article and gave final approval for the version to be published. HV and PV designed the study, reviewed the article, and gave final approval for the version to be published. All authors contributed to the article and approved the submitted version.

## Author Disclaimer

The views expressed are those of the author(s) and not necessarily those of the NHS, the NIHR or the Department of Health and Social Care.

## Conflict of Interest

The authors declare that the research was conducted in the absence of any commercial or financial relationships that could be construed as a potential conflict of interest.

## Publisher's Note

All claims expressed in this article are solely those of the authors and do not necessarily represent those of their affiliated organizations, or those of the publisher, the editors and the reviewers. Any product that may be evaluated in this article, or claim that may be made by its manufacturer, is not guaranteed or endorsed by the publisher.

## Abbreviations

BT: backward translation
CE: cultural effect
DSM: diagnostic and statistical manual of mental disorders
EGPRN: European general practice research network
FPs: family practice physicians
FT: forward translation
PRISMA: preferred reporting items for systematic reviews and meta-analyses
RAND: research and development
RAND/UCLA: research and development/University of California Los Angeles.
